# Preparation of novel Au-Nb_3_O_7_F nanosheets for the photodegradation of tetracycline hydrochloride

**DOI:** 10.3389/fchem.2024.1412457

**Published:** 2024-05-22

**Authors:** Zhiyuan Wang, Li Ren, Zhi Chen, Yao Chen, Xin Tian, Guoying Wei

**Affiliations:** College of Materials and Chemistry, China Jiliang University, Hangzhou, China

**Keywords:** niobium oxyfluoride, gold nanoparticles, tetracycline hydrochloride, visible light, photodegradation

## Abstract

Water pollution caused by antibiotics is a growing problem and photodegradation by efficient catalysts is an environmentally friendly technology that can effectively degrade organic pollutants in water. Here, a novel method was innovatively used to synthesize niobium oxyfluoride (Nb_3_O_7_F) nanosheets decorated with Au nanoparticles, which is the first report for the composites of Au and Nb_3_O_7_F. We prepared the Nb_3_O_7_F nanosheets via hydrothermal synthesis followed by deposition of Au nanoparticles on their surface using HAuCl_4_. The prepared samples were characterized by XRD, HRTEM, XPS, and UV–Vis. The diameters of most Au NPs are ranging from 5 to 25 nm with an average size of about 16.9 nm, as well as the Nb_3_O_7_F nanosheets in size ranging from 200 nm to 700 nm. The chemical composition of the Au-Nb_3_O_7_F showed a Au/Nb atomic ratio of 1/10, as well as a Nb/O/F ratio of 3/7/1. UV–Vis spectrum reveals a largest absorption peak at 520 nm for the Au-Nb3O7F nanosheets. The prepared Au-Nb_3_O_7_F nanomaterials were applied to the visible-light photodegradation of tetracycline hydrochloride, with the photocatalytic degradation rate reached more than 50% under the optimal conditions within 1 h. Capture experiments indicated that h^+^ and •O_2_^-^ are the main active substances involved during the course of the photodegradation. Furthermore, the proposed mechanism for the photodegradation of the novel Au-Nb3O7F nanosheets was given.

## Introduction

Development of new efficient photocatalysts addressed to environmentally friendly processes, aiming at the elimination of organic pollutants and transformations of waste is still an emerging issue (Yang et al., 2021a; Shurbaji et al., 2021; Zhang Y. et al., 2022; De Ilurdoz et al., 2022; Balakrishnan et al., 2023; Li et al., 2023; Lanjwani et al., 2024; Vinayagam et al., 2024). Environmentally friendly semiconductors, such as TiO_2_, ZnO, Nb_2_O_5_, are efficient heterogeneous photocatalysts (Lang et al., 2014; Ani et al., 2018; Anwer et al., 2019; Wetchakun et al., 2019; Jiang et al., 2020; Zhao et al., 2020). In most cases, these semiconductors only respond to UV light irradiation and cannot be efficiently excited under visible light due to their wide band gaps (*ca*. 3.2 eV for TiO_2_, ZnO, and Nb_2_O_5_), leading to limited utility for photosynthesis and photodegradations. According to the literature, one of the effective methods to improve their activity under visible light irradiation and achieve this goal is to design more complex catalytic systems and construct heterojunction containing plasmonic metals, in order to broaden the absorption spectrum as well as to improve the carrier separation efficiency (Leong et al., 2018; Prakash et al., 2018; Yang et al., 2021b; Gao et al., 2022; Liu et al., 2022; Ly et al., 2023; Mishra et al., 2023). Up to now, many researchers have reported that these large band gap semiconductors combined with noble metal nanoparticles (such as Au, Ag, *etc*.) are effective photocatalysts for photoelectrochemical water splitting and photocatalytic degradation of organic dyes and pollutants (Leong et al., 2018; Prakash et al., 2018; Ly et al., 2023).

Nb_3_O_7_F is a metal-oxyfluoride semiconductor with a band gap of *ca.* 2.9–3.2 eV, as well as low cost and high chemical stability. Nb_3_O_7_F is a commonly used material for a variety of applications including catalysts, sensors, recording materials, electrochemical supercapacitors, Li-ion batteries, and field emission materials (Idrees et al., 2014; Hirai et al., 2018; Chen et al., 2021; Li et al., 2021; Zhang et al., 2021). However, Au combined with Nb_3_O_7_F had been never investigated before, including photocatalytic degradation of contaminants in water. Furthermore, tetracycline is one of the mostly occurring synthetic antibiotics and is commonly used, leading to a growing problem of water pollution. As a result, degradation of such highly stable antibiotic is much needed (Xiao et al., 2020; Qin et al., 2021; Zhang J. et al., 2022; Huang et al., 2022; Zhao L. et al., 2023; Umair et al., 2023; Yuan et al., 2023). Common methods like adsorption on activated carbons and reverse osmosis are adopted for this purpose. However, these are not effective in destructing and demolishing the contaminants. Ozonation and chlorination are other methods for antibiotics degradation and contaminants elimination from water but they are considered as expensive and inefficient to use (Choi et al., 2008; Sánchez-Polo et al., 2015; Su et al., 2020; Calcio Gaudino et al., 2021; Lu et al., 2021). Hence there is a need of the production of such photocatalyst, which are less toxic and more effective as well as can easily degrade these contaminants from water.

Herein, we successfully synthesized a kind of novel Au-Nb_3_O_7_F composite nanosheets for the first time, which could be used in the visible light photocatalytic degradation of tetracycline hydrochloride. The Nb_3_O_7_F nanosheets were prepared *via* different methods followed by the deposition of gold nanoparticles on their surface using HAuCl_4_. The prepared nanosheets were characterized by XRD, HRTEM, XPS, and UV–Vis. The composite nanoparticles of Nb_3_O_7_F nanosheets decorated with gold nanoparticles demonstrate distinct and robust surface plasmon resonance (SPR) effects within the visible light range. Photocatalytic activity of the nanomaterials was evaluated under visible light irradiation and demonstrated that the decoration of the gold nanoparticles with the Nb_3_O_7_F nanosheets led to an enhancement of the photocatalyst performance. Furthermore, preliminary mechanistic studies indicated that h^+^ and •O_2_^−^ are the main active substances in the degradation of TC-HCl.

## Materials and methods

### Materials

Chemicals such as niobium nanopowder (Nb, 99.9%, 60–80 nm), anatase titanium dioxide (TiO_2_, 99.8%, 60 nm), zinc oxide (ZnO, 99.9%, 30 ± 10 nm), niobium pentoxide (Nb_2_O_5_, 99.9%, AR), *tert*-butanol (C_4_H_10_O, TBA, >99.0%, AR), ethylene diamine tetraacetic acid disodium salt dihydrate (C_10_H_14_N_2_Na_2_O_8_.2H_2_O, EDTA·2Na, 98%, AR), sodium sulfate (Na_2_SO_4_, AR), tetracycline hydrochloride (C_22_H_24_N_2_O_8_·HCl, TC-HCl, BR), polyvinyl pyrrolidone ((C_6_H_9_NO)_n_, PVP, Average Mw: 8000, K16-K18, AR), *L*-ascorbic acid (C_6_H_8_O_6_, >99.0%, AR), hydrofluoric acid (HF, 40%, AR), Nafion (D520, 5wt% in mixture of lower aliphatic alcohols) had been all purchased from Macklin Chemical *Co., Ltd*. Ethanol (C_2_H_6_O, 99.7%, AR), 2,2,6,6-tetramethylpiperidinooxy (C_9_H_18_NO, TEMPO, 98%, AR) had been all purchased from Hangzhou Shuanglinchem *Co., Ltd*. Chloroauric acid (HAuCl_4_, 99%, AR) was purchased from CIVI(Shanghai). Ultrapure water was used in the process of all the experiments. All chemical reagents have been used as received without any further purification.

### Synthesis of Nb_3_O_7_F nanosheets

The Nb_3_O_7_F nanosheets were synthesized through a novel simple hydrothermal method. Niobium nanopowder (diameter: 60–80 nm, 0.500 g, 5.38 mmol), hydrofluoric acid (1.000 mL, *w*/*w*: 40%) were added into 50.0 mL deionized-water and stirred. After stirring for 2 h at room temperature, the solution was transferred into a Teflon-lined stainless steel autoclave for a hydrothermal reaction at 160°C for 12 h. The samples were cooled down to room temperature, centrifuged, and washed with deionized water and ethanol. This grey sample was dried and labeled as Nb_3_O_7_F nanosheets.

### Synthesis of Au-Nb_3_O_7_F nanosheets

The Au NPs were prepared by the chemical reduction method in a previous report (Leung et al., 2012). 0.100 g Nb_3_O_7_F nanosheets, 0.700 g Polyvinyl pyrrolidone, 1.200 g *L*-ascorbic acid was added into a 100 mL round-bottomed flask. Put it into an oil bath and heated until 90°C. Then a certain volume of HAuCl_4_ aqueous solution (0.050 g/mL) was added into the mixture. The color of the solution turns into wine red in the boiling state for 3 h. Finally, the sample was cooled down to room temperature, centrifuged, washed with deionized water and ethanol for three times respectively and putted into an oven at 60°C for 12 h. The dark-red solid sample was labeled as xAu-Nb_3_O_7_F (x refers to the mass ratio of HAuCl_4_ and Nb_3_O_7_F nanosheets, the exact amounts of HAuCl_4_ for each synthesis are shown in Table 1).

**TABLE 1:** Exact amounts of HAuCl_4_ for each synthesis.

x	HAuCl_4_ (mL)
0.1	0.200
0.2	0.400
0.4	0.800

### Sample characterization

X-ray diffraction (XRD) patterns were obtained on a Rigaku Ultima IV in the range 2θ was from 10° to 90° with the scan speed of 5°/min to identify the crystalline structure of Au-Nb_3_O_7_F nanosheets with graphite monochromatic Cu−Kα radiation (*λ* = 0.154178 nm). Scanning electron microscope (SEM) images and energy dispersive spectroscopy (EDS) spectra were captured using a Hitachi S-4800. For transmission electron microscopy (TEM) analysis, a drop of nanosheet dispersion in deionized water was dropped onto a nonfixed carbon-coated copper grid. Ultra high resolution transmission electron microscopy (HRTEM) images were acquired using a JEM-2100F with an accelerating voltage of 200 kV. X-ray photoelectron spectroscopy (XPS, Thermo Scientific K-Alpha) with a monochromatic Al Kα radiation was used to analyze the elemental composition on the surface and oxidation state of material. The ultraviolet−visible absorption spectrum was measured using Shimadzu UV-3600 integrating sphere.

### Photoelectrochemical measurements

A 300 W Xe lamp assembled with a cutoff filter (λ > 400 nm) was used as the light source in the photocatalytic degradation experiment. First, 10 mg of catalyst is added to 50 mL of a certain concentration tetracycline hydrochloride (TC-HCl) solution in a beaker. Before the light irradiation, the solution with the photocatalyst was magnetically stirred in darkness for 1 h in order to establish adsorption equilibrium. During the light irradiation, 3.5 mL of the solution was taken out every 10 min and centrifuged to separate the solid. The concentration of TC-HCl was investigated at its maximum adsorption of 356 nm on the UV 2600 spectrophotometer. The photocatalytic degradation rate was then calculated. The photodegradation rate (DR) (%) of TC-HCl by the catalyst in this experiment was determined by using the following equation:

$$\text{DR}=\left(1‐\frac{A_{i}}{A_{0}}\right)\times 100\%$$

where A_i_ is the absorbance of the sample at the time of irradiation and A_0_ is the initial absorbance of the reactive degradates after reaching adsorption-desorption equilibrium.

Electrochemical tests were operated at the CHI660E electrochemical workstation. By using a standard three-electrode cell with a platinum wire as the counter electrode, a standard calomel electrode (SCE) as the reference electrode, and 10 mg of prepared Nb_3_O_7_F or 0.4 Au-Nb_3_O_7_F were separately dissolved in 1.5 mL of Nafion solution and coated on the FTO conducting glass (1 cm × 1 cm) by spin coating to form the working electrode, respectively. Forty milliliters of Na_2_SO_4_ (0.5 M) was used as an electrolyte and a 300 W Xe lamp assembled with a cutoff filter (λ > 400 nm) was used as the light source.

## Results and discussion

### Synthesis and characterization of Au-Nb_3_O_7_F nanoparticles

**FIGURE 1:**
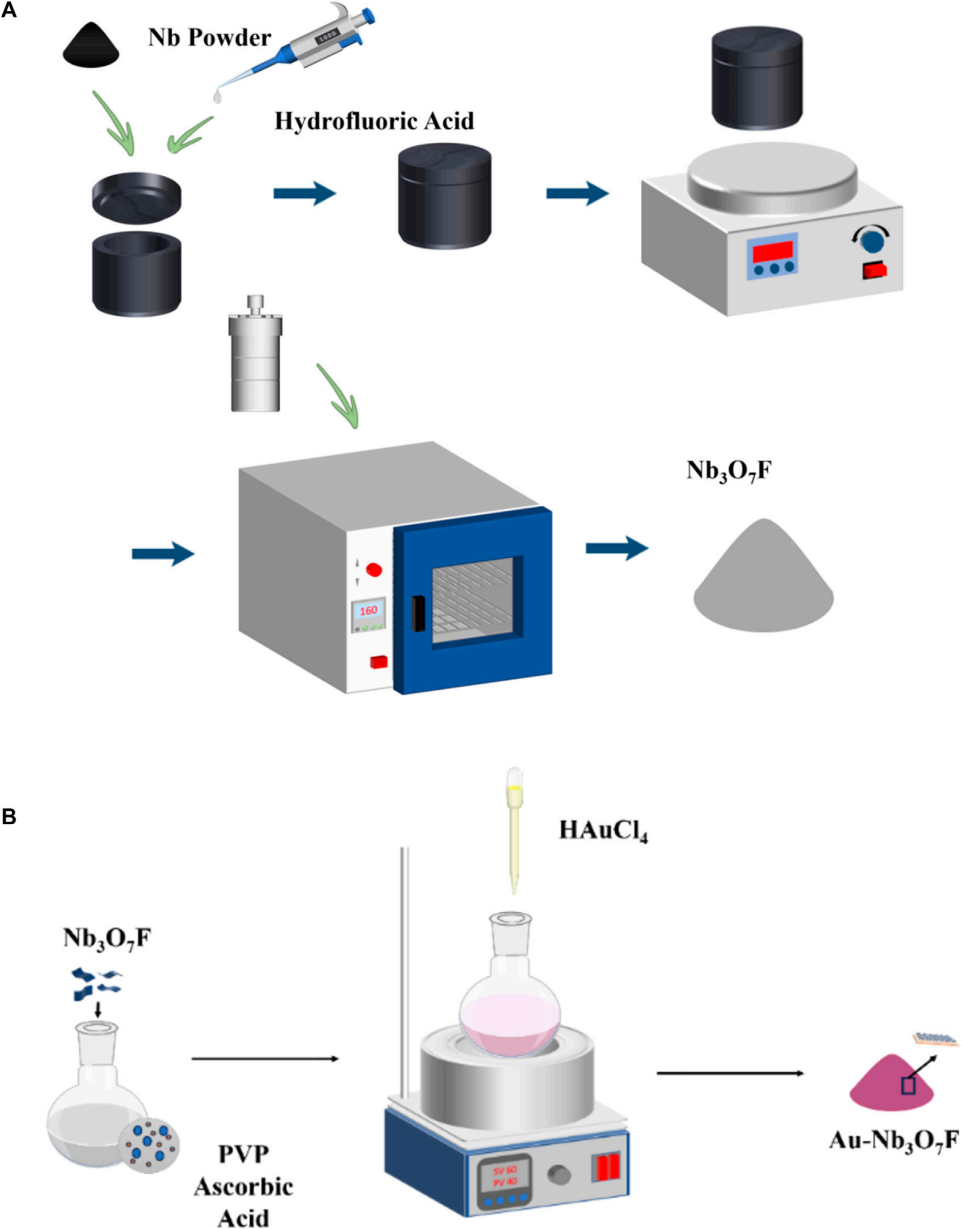
The synthesis process of **(A)** Nb_3_O_7_F nanosheets and **(B)** Au-Nb_3_O_7_F nanosheets.

A schematic diagram of the synthesis process of Nb_3_O_7_F nanosheets and Au-Nb_3_O_7_F nanosheets is shown in Figure 1. These Au-Nb_3_O_7_F nanosheets were synthesized by a combined process of niobium nanopowder hydrothermal process and followed by a reduction process of HAuCl_4_ to form the gold nanoparticles to deposit on the Nb_3_O_7_F nanosheets.

**FIGURE 2:**
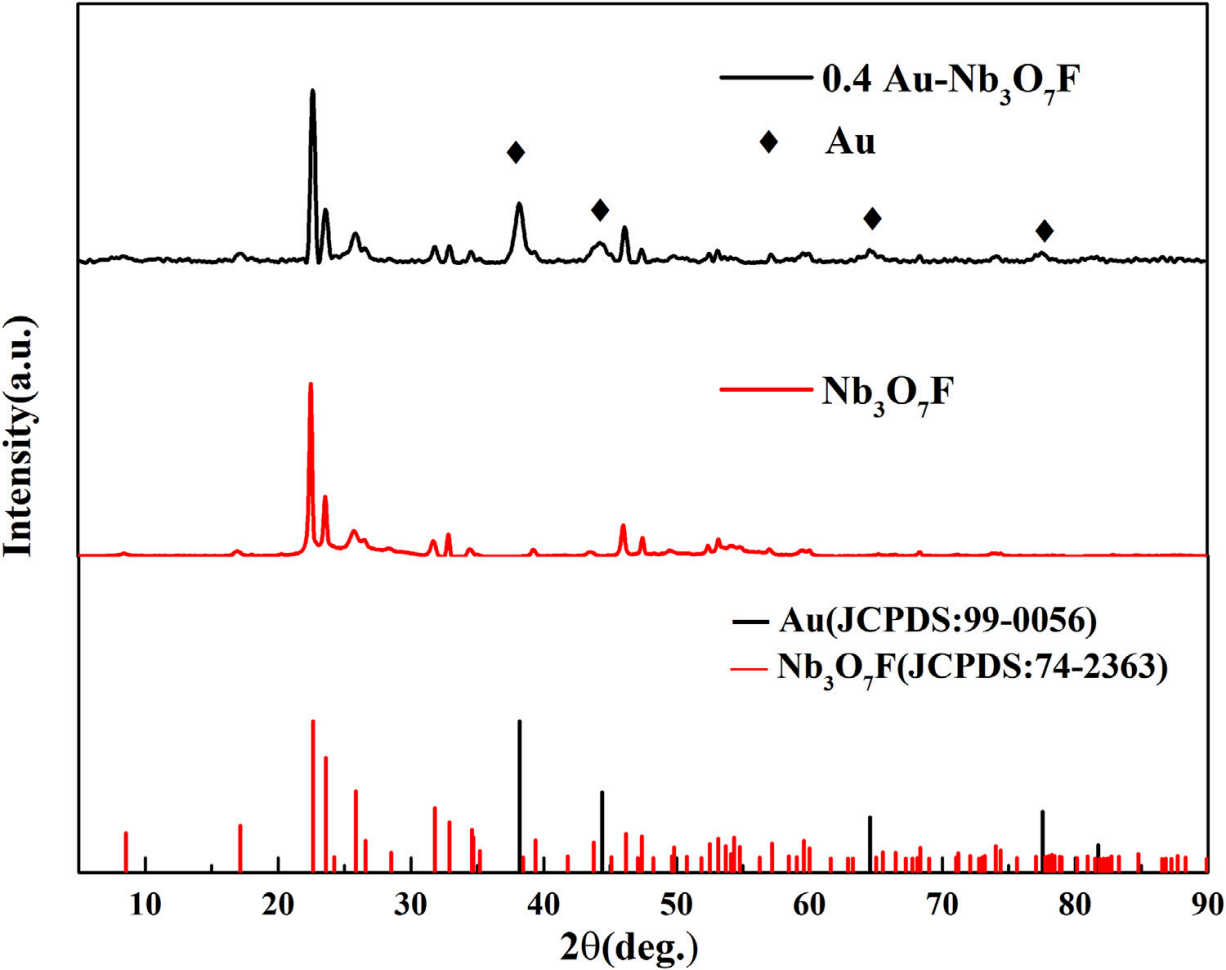
XRD patterns of the sample 0.4 Au-Nb_3_O_7_F.

Powder X-Ray Diffraction (XRD) was used to detect the crystal phase of the Au and Nb_3_O_7_F composite nanostructure. XRD diagrams for Au-Nb_3_O_7_F nanosheets and Nb_3_O_7_F nanosheets are presented in Figure 2, which shows that the XRD pattern accurately corresponds to monoclinic phase of Nb_3_O_7_F and Au, respectively. Peaks at 2θ values of 17.15°, 22.62°, 23.59°, 25.85°, 31.81°, 32.91°, 34.69°, 47.39°, 53.13°, and 84.76°, *etc*., corresponding to the Nb_3_O_7_F (JCPDS No. 74-2363), and the strong diffraction peaks observed also suggest that the product’s crystallinity is high. In the image we can also observe the reflection peak of Au NPs at 38.18°, 44.38°, 64.57°, and 77.56°, correspond to the peaks of Au (JCPDS No. 99-0056) on the Nb_3_O_7_F, which is weaker than the peaks correspond to Nb_3_O_7_F, without any detectable diffraction peaks of NbO_2_ or any other crystalline phase, thereby indicating that the product is the Au-Nb_3_O_7_F nanosheets.

**FIGURE 3:**
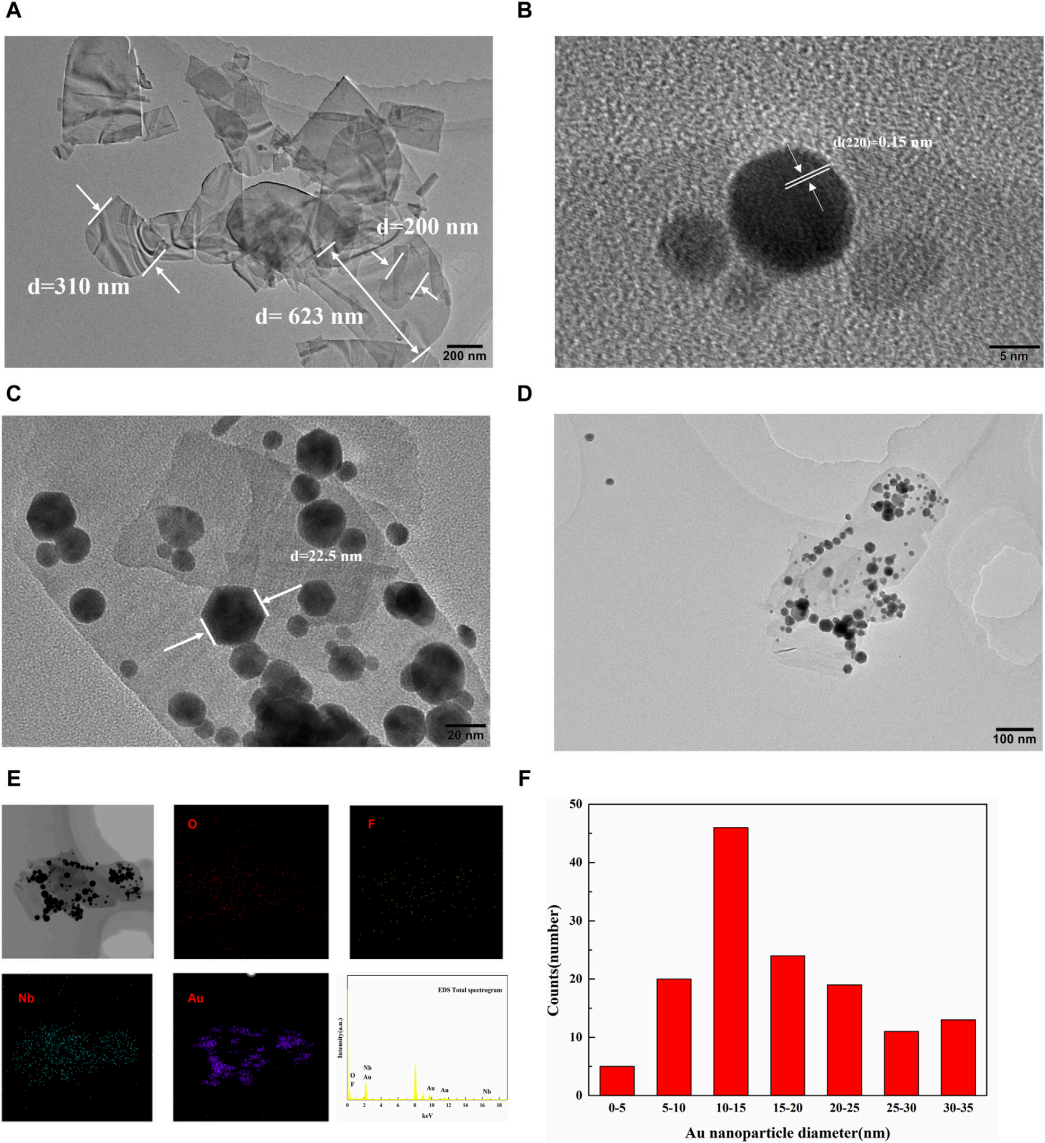
**(A)** HRTEM images of Nb_3_O_7_F; **(B–D)** HRTEM images of 0.4 Au-Nb_3_O_7_F; **(E)** EDS Mapping image of 0.4 Au-Nb_3_O_7_F; **(F)** histogram of the size distribution of Au NPs.

The high resolution transmission electron microscopy (HRTEM) images of the Nb_3_O_7_F and the Au-Nb_3_O_7_F composites are shown in Figures 3A–D, respectively, indicated that the sample is in size ranging from 200–700 nm. These Au nanoparticles were successfully loaded on the surface of the Nb_3_O_7_F nanoparticles and the deposition of Au NPs does not change the original appearance of Nb_3_O_7_F nanosheets (Figures 3B–D). There are Au NPs on the surface of Nb_3_O_7_F nanosheets. Furthermore, energy dispersive spectroscopy (EDS) characterizations suggested that the sample contained Au, Nb, F, and O signals (Figure 3E). The EDS mapping images give the elemental distribution of the Nb_3_O_7_F nanosheets, which clearly proves that Nb, O, and F elements are uniformly distributed on these nanosheets, with the Au element distributed on these nanosheets as the nanoparticles. The HRTEM image indicates that the lattice spacings of 0.15 nm correspond to the (220) crystal planes of Au (Figure 3B). The diameter of most Au NPs on the surface of the nanosheets are ranging from 5 nm to 25 nm with an average size of about 16.9 nm (Figure 3F).

**FIGURE 4:**
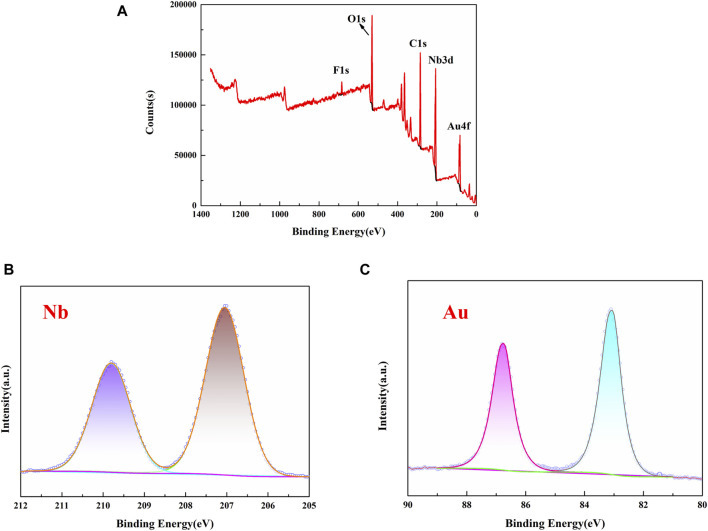
**(A)** XPS full spectrum of 0.4 Au-Nb_3_O_7_F; XPS fine spectra of **(B)** Nb 3d, and **(C)** Au 4f of 0.4 Au-Nb_3_O_7_F.

X-ray photoelectron spectroscopy (XPS) was used to analyze the composition and oxidation state of the atoms in the Au-Nb_3_O_7_F nanosheets. As shown in Figure 4A, the survey spectrum proves the existence of Au, Nb, O, and F elements. The peak at 684.3 eV is typically attributed to F 1s (Li et al., 2018). The peak at 530.2 eV is typically attributed to O 1s (Li et al., 2018). The oxidation state of the nanosheets are indicated that the nanosheets are composed of oxygen. The oxidation state of niobium, present as Nb^5+^, were differentiated by peak fitting of the Nb 3d spectra and the spectra of Nb 3d are shown in Figure 4B. The predominant peaks located at lower binding energies of 207.1 eV, was classified as characteristic of Nb 3d_5/2_. The higher binding energies of 209.8 eV are characteristic of the Nb 3d_3/2_ spin-orbit components. Two peaks were attributed to Nb^5+^ 3d (207.1 and 209.8 eV) (Li et al., 2018). Above results confirm the existence of Nb-F bond and Nb-O band (Li et al., 2018). The XPS of Au 4f_5/2_ and Au 4f_7/2_ are also shown in Figure 4C. The main peaks located at BEs of 83.1 and 86.8 eV representing metallic Au (Au^0^) (Dong et al., 2019; Lin et al., 2020). Based on these results, it can be confirmed that Au-Nb_3_O_7_F has been synthesized successfully. In addition, the XPS spectrum shows a Au/Nb atomic ratio of 1/10, as well as the nanosheets have a Nb/O/F atomic ratio of 3/7/1 (see Supplementary Table S2).

**FIGURE 5:**
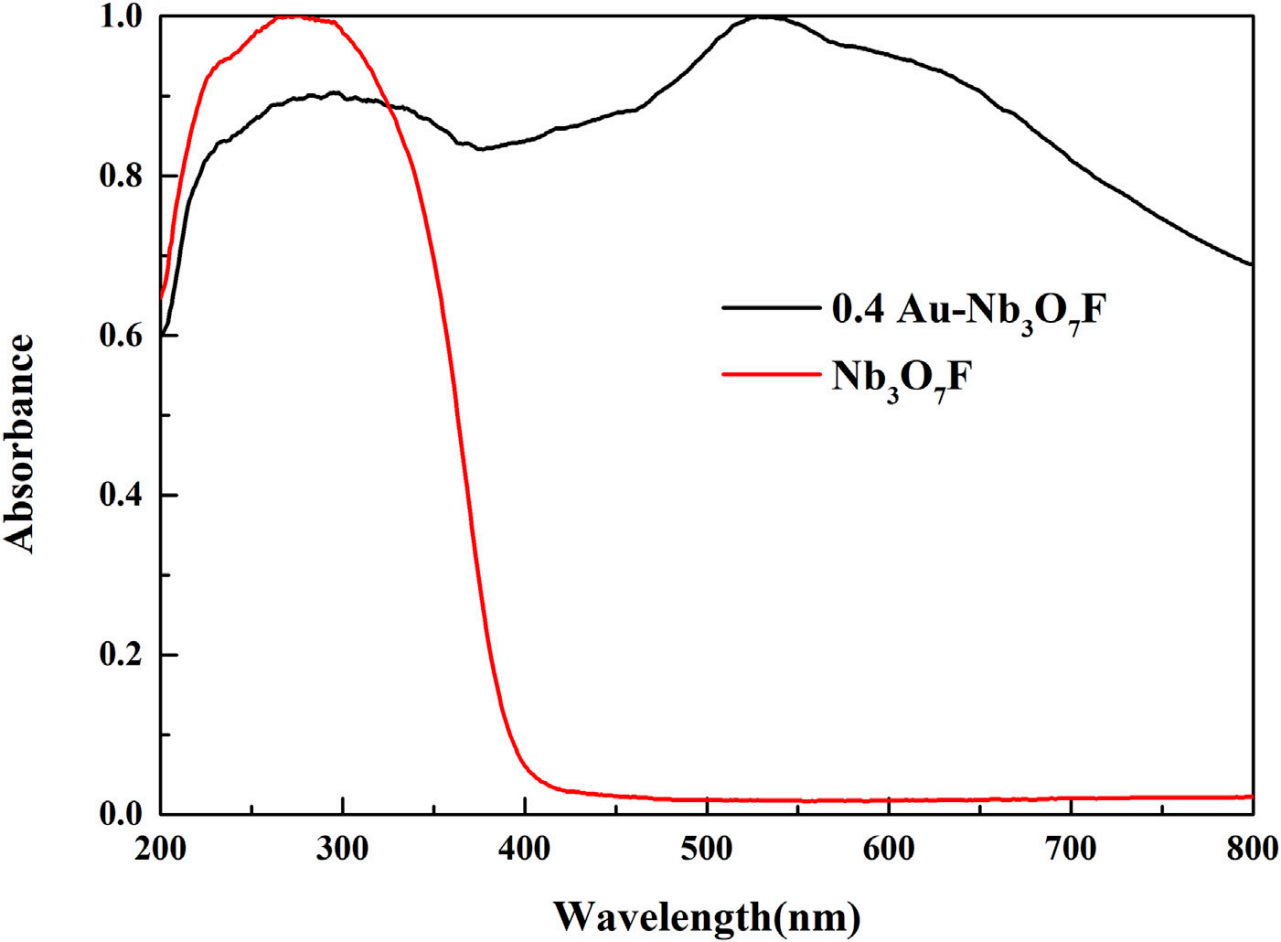
UV-Vis DRS spectra of 0.4 Au-Nb_3_O_7_F and Nb_3_O_7_F.

The photoresponse range of the catalyst is one of the important factors affecting the photocatalytic effect. The ultraviolet−visible (UV−Vis) absorption spectrum of 0.4 Au-Nb_3_O_7_F and Nb_3_O_7_F with wavelengths ranging from 200 nm to 800 nm were displayed in Figure 5. It can be seen from the figure that the Nb_3_O_7_F nanosheets without Au NPs mainly absorbs light less than 425 nm. However, the optical absorption of 0.4 Au-Nb_3_O_7_F nanosheets increases ranging greater than 400 nm in visible light region, as well as the largest absorption peak at 520 nm. It reveals a broad absorption ranging from 400 nm to 800 nm for the Au-Nb_3_O_7_F nanosheets. This optical phenomenon is likely attributed to the LSPR effect, which is believed to be closely related to the Au nanoparticles, and the largest absorption peak at 520 nm of 0.4 Au-Nb_3_O_7_F nanosheets was attributed to the Au NPs. The 0.4 Au-Nb_3_O_7_F nanosheets possess strong LSPR effect and are expected to an excellent candidate for visible light photodegradation (Cui et al., 2022; Guo et al., 2023; Zhao et al., 2024). In addition, the bandgap calculated by the Tauc’s plot is shown in Supplementary Figure S8. The band gaps of 0.4 Au-Nb_3_O_7_F nanosheets and Nb_3_O_7_F nanosheets are 2.38 eV and 3.28 eV, respectively, indicating that the forbidden bandwidth of the 0.4 Au-Nb_3_O_7_F became narrower than that of Nb_3_O_7_F, and the narrower forbidden bandwidth made the catalysts easier to be excited, resulting in a better photocatalytic effect.

### Photocatalytic properties of the Au-Nb_3_O_7_F nanosheets

**FIGURE 6:**
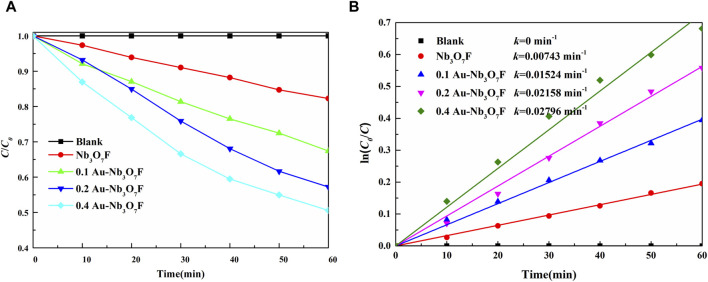
Photodegradation curves of tetracycline hydrochloride **(A)** photodegradation versus time curves; **(B)** photodegradation rate curves.

The degradation ability of the prepared photocatalysts was examined using tetracycline hydrochloride as the target pollutant. Figure 6 shows the graphs of the degradation of tetracycline hydrochloride under the same conditions for Nb_3_O_7_F nanosheets without gold nanoparticles, as well as the Au-Nb_3_O_7_F nanosheets with different ratios of gold nanoparticles. In Figure 6A, without any catalyst, the concentration of the tetracycline hydrochloride was almost not changed after 1 h of visible-light irradiation, and the degradation effect of Nb_3_O_7_F nanoparticles without Au NPs was the worst, with only 18.0% degradation after 1 h of visible-light irradiation. The degradation effect of the Nb_3_O_7_F nanosheets combined with any ratio of gold nanoparticles was better than that of the single Nb_3_O_7_F nanosheets, and the degradation effect was related to the mass ratio of the gold nanoparticles and the Nb_3_O_7_F nanosheets. The photocatalytic degradation rate of the prepared 0.4 Au-Nb_3_O_7_F composites reached 50.6% within 1 h. The degradation efficiency showed an increasing trend with the increasing loading of gold nanoparticles, which might be attributed to the fact that the loading of gold nanoparticles would improve the effect of the photocatalytic degradation. The degradation of tetracycline hydrochloride by all the photocatalysts was in accordance with the first order kinetic equation. Figure 6B shows the first-order kinetic fitting curves of Nb_3_O_7_F nanosheets, 0.1 Au-Nb_3_O_7_F, 0.2 Au-Nb_3_O_7_F and 0.4 Au-Nb_3_O_7_F, with rate constants of 7.43 × 10^−3^, 1.52 × 10^−2^, 2.16 × 10^−2^, and 2.80 × 10^−2^ min^-1^, respectively, and the reaction rate constant of 0.4 Au-Nb_3_O_7_F nanosheets was about 4 times as high as those of Nb_3_O_7_F nanosheets. HPLC was used to monitor whether the byproducts or intermediates were formed during the photocatalytic degradation of 0.4 Au-Nb_3_O_7_F nanosheets. The test results are shown in the Supplementary Material.

The reusability/sustainability of photocatalysts plays an important role in their practical usage. The selected 0.4 Au-Nb_3_O_7_F nanosheets were collected and washed after 1 h of the photocatalytic process and operated repeatedly for another three cycles according to the same procedures (see Supplementary Figure S4). However, the activity is significantly reduced at the 3rd cycle, which may result from the aggregation and dissociation of the gold nanoparticles from the Nb_3_O_7_F nanosheets. Additionally, aggregations of the gold nanoparticles are presented in the HRTEM image of 0.4 Au-Nb_3_O_7_F nanosheets after the 3rd cycle of the photocatalytic reaction (see Supplementary Figure S5). These features as well as the mass loss during the photodegradation process and the recycle process may contribute for the significant decrease of photocatalytic efficiency in the 3rd cycle.

**FIGURE 7:**
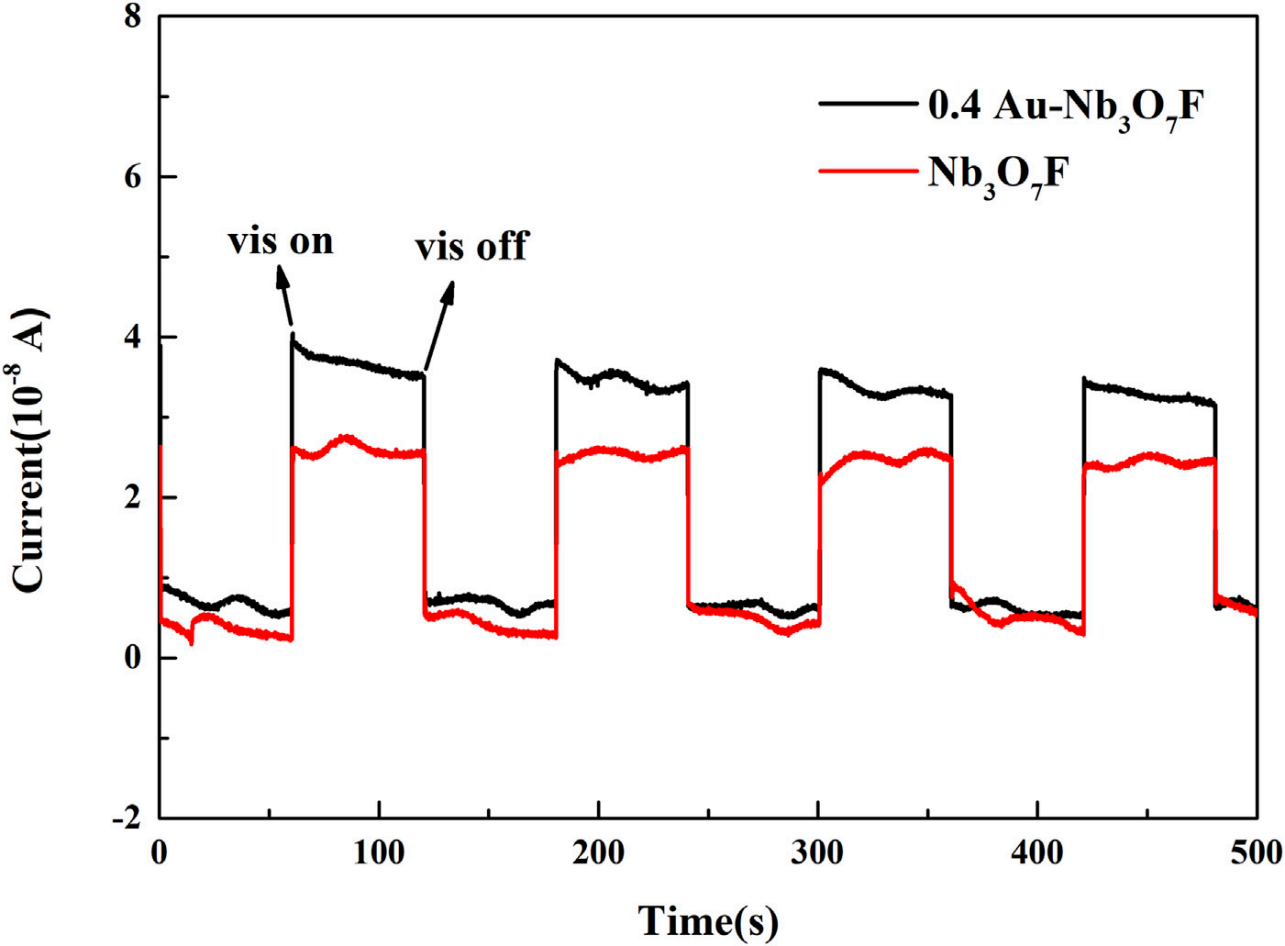
Transient photocurrent response test curves for Nb_3_O_7_F and 0.4 Au-Nb_3_O_7_F nanosheets.

The photocurrent at the light on/off conversion is a robust approach to analyzing the separation/transport of the photogenerated carriers, which is closely connected with the photocatalytic performance. Figure 7 shows the photocurrent response of Nb_3_O_7_F nanosheets and 0.4 Au-Nb_3_O_7_F nanoparticles under visible irradiation. Obvious photocurrents are present in both the photocatalysts when the irradiation is turned on, and 0.4 Au-Nb_3_O_7_F nanosheets have the greater photocurrent compared to Nb_3_O_7_F. This indicates that the 0.4 Au-Nb_3_O_7_F nanosheets may have the higher separation/migration efficiency of photoexcited carriers compared with Nb_3_O_7_F nanosheets without gold nanoparticles. This finding indicates that the combination of the Au nanoparticles as well as the Nb_3_O_7_F nanosheets may be benefit for the charge separation, leading to the improved photocatalytic performance (Liang et al., 2020; Guo et al., 2023).

**FIGURE 8:**
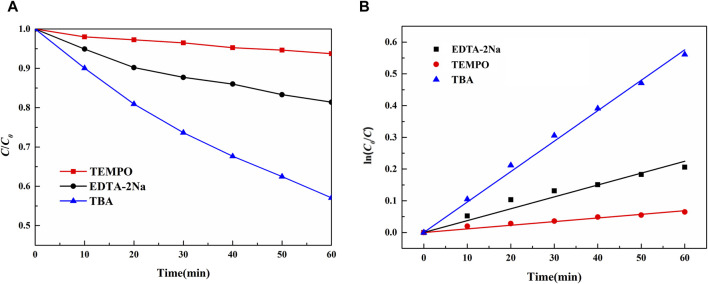
Photodegradation of tetracycline hydrochloride with different radical scavengers. **(A)** photodegradation versus time curves; **(B)** photodegradation rate curves.

Free radical capture experiments were used to investigate the active substance of 0.4 Au-Nb_3_O_7_F nanosheets in photocatalytic reactions. *Tert*-butanol (TBA), ethylene diamine tetraacetic acid disodium salt (EDTA·2Na), and TEMPO were added to the aqueous TC-HCl solution to capture the ·OH, h^+^, and ·O_2_^−^ produced during the photodegradation process, respectively (Lang and Zhao, 2018; Zhang B. et al., 2022; Feng et al., 2022). From Figure 8, it can be seen that EDTA·2Na and TEMPO have a greater effect on the photocatalytic degradation effect, while TBA has little effect on the photocatalytic degradation effect, which proves that h^+^ and ·O_2_^−^ are the main active substances in the degradation of TC-HCl by 0.4 Au-Nb_3_O_7_F nanosheets (Guo et al., 2019; Zhao Y. et al., 2023; Yang et al., 2023).

**FIGURE 9:**
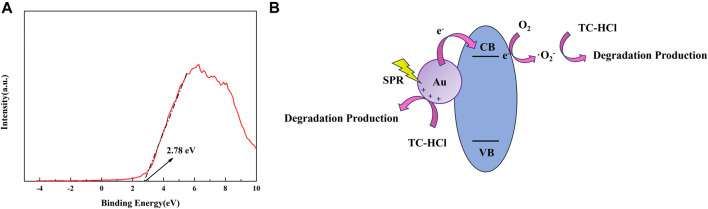
**(A)** XPS valence band spectra **(B)** Plausible mechanism of the Au-Nb_3_O_7_F nanosheets.

According to the kinetic study, the presence of Au nanoparticles was able to increase the apparent rate constant of the reaction by 4 folds (Figure 6), which might be attributed to the fact that the loading of gold nanoparticles would improve the effect of the photocatalytic degradation. In order to determine the conduction band and valence band positions, the valence band spectra was obtained by XPS test, as shown in Figure 9A, and the valence band position of Nb3O7F was 2.54 eV (Kamat, 2002) (VB(Vs NHE)=Ф+2.78-4.44 =4.2+2.78-4.44 =2.54 eV, see ref. (Feng et al. 2020), indicating that the conduction band position of Nb_3_O_7_F was higher than the fermi level of gold nanoparticles, since the fermi level of gold is more positive (0.75 V) (Kamat, 2002). Combining the above data and previous reports, the photocatalytic mechanism for the photodegradation of the novel Au-Nb3O7F nanosheets was outlined in Figure 9(b). It is suggested that the most plausible mechanism for the plasmonic enhancement of photocatalytic in this study is via charge transfer and local electric field enhancement. Previous reports have suggested that when exposed to visible light radiation, the SPR process excites electrons in Au, which could then be transferred to the conduction band of the adjacent semiconductor Tian and Tatsuma, 2005; Mubeen et al., 2011; Khalil et al., 2019. Furthermore, the presence of SPR can also generate intense local electric fields creating “hot spot” regions near the surface of Au nanoparticles Khalil et al., 2019; Le et al. 2008. As a result, the population of electrons in the “hot spot” regions that can be migrated to CB of the adjacent semiconductor is also increased, which are essential for photocatalytic reactions. With electron transfer from the Au nanoparticles fill into the conduction band (CB) of Nb3O7F, leading to the formation of superoxide radicals, and the degradation of TC-HCl could occur. In addition, when exposed to visible light radiation, the positive hole (or positive charge) left in gold nanoparticles would get electrons from the TC-HCl, with the degradation of TC-HCl could occur.

## Conclusion

In summary, we have successfully innovatively synthesized a kind of novel composite nanosheets composed of Au nanoparticles and Nb_3_O_7_F for the first time, which could be used for the photocatalytic degradation of tetracycline hydrochloride. The niobium oxyfluoride (Nb_3_O_7_F) nanosheets were prepared *via* hydrothermal synthesis, followed by deposition of gold nanoparticles on their surface. The hybrid nanosheets of Nb_3_O_7_F combined with gold nanoparticles demonstrate surface plasmon resonance (SPR) effects within the visible light range. Compared with Nb_3_O_7_F nanosheets without gold nanoparticles, the Au-Nb_3_O_7_F composite nanosheets have better photocurrent response efficiency, and exhibit better photocatalytic performance in the visible-light degradation of tetracycline hydrochloride, which could be applied in the treatment of organic pollutants in water.

## Data Availability

The original contributions presented in the study are included in the article/Supplementary Material, further inquiries can be directed to the corresponding authors.
