# Supplementary material for: Preparation of novel Au-Nb3O7F nanosheets for the photodegradation of tetracycline hydrochloride
Source: Front Chem. 2024 May 22;12:1412457. doi: 10.3389/fchem.2024.1412457 (PMC11165350; doi:10.3389/fchem.2024.1412457)
Supplement: Supplementary file 1 [file DataSheet1.docx]

Supplementary Material

**Preparation of Novel Au-Nb_3_O_7_F Nanosheets for the**

**Photodegradation of Tetracycline Hydrochloride**

Zhiyuan Wang, Li Ren*, Zhi Chen, Yao Chen, Xin Tian, Guoying Wei*

College of Materials and Chemistry, China Jiliang University, Hangzhou, China

**Page S2: Comparisons to other photocatalytic systems**

**Page S3: Photoluminescence spectra of 0.4 Au-Nb_3_O_7_F and Nb_3_O_7_F**

**Page S4: HPLC of 0.4 Au-Nb_3_O_7_F degradation of TC-HCl**

**Page S7: Recyclability of 0.4 Au-Nb_3_O_7_F nanosheets degradation of TC-HCl**

**Page S8: Photodegradation curves of tetracycline hydrochloride for 4 h**

**Page S9: Chemical composition of the Au/Nb_3_O_7_F composites**

**Page S10: Photodegradation curves of TC-HCl concerning the adsorption equilibrium**

**Page S11: Tauc’s Plot**

**I. Comparisons to other photocatalytic systems**

The photodegradation curves of tetracycline hydrochloride compared with Nb_2_O_5_, ZnO, and TiO_2_ below. The photocatalytic degradation was compared with commercially available photocatalysts such as TiO2, ZnO, Nb2O5. The photocatalytic degradation is shown in **Supplementary Figure 1**, which shows after 1 h of visible-light irradiation, the degradation effect of the TiO_2_, ZnO, and Nb_2_O_5_ nanoparticles were 42.1%, 34.8%, and 6.8%, respectively, which is lower than the degradation efficiency of 0.4 Au-Nb3O7F. As a result, most of the photocatalysts could be separated from the mixture after the photocatalytic degradation by centrifugation, so potential environmental implications would be possible.

**Supplementary Table 1. Photodegradation curves of tetracycline hydrochloride compared with commercially available Nb_2_O_5_, ZnO, and TiO_2_**


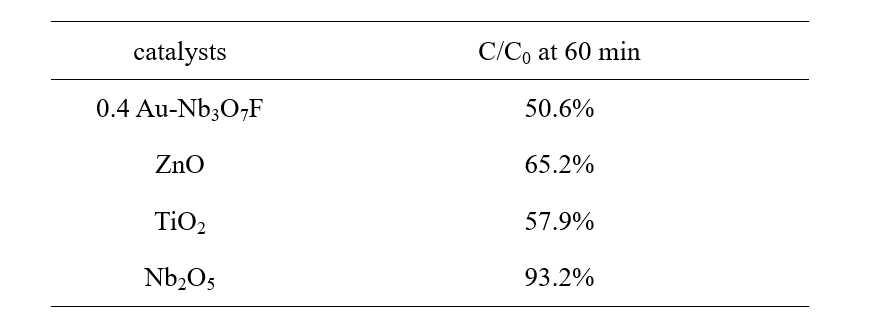





**Supplementary Figure 1. Photodegradation curves of tetracycline hydrochloride compared with commercially available Nb_2_O_5_, ZnO, and TiO_2._**

**II. Photoluminescence spectra of 0.4 Au-Nb_3_O_7_F and Nb_3_O_7_F**

**
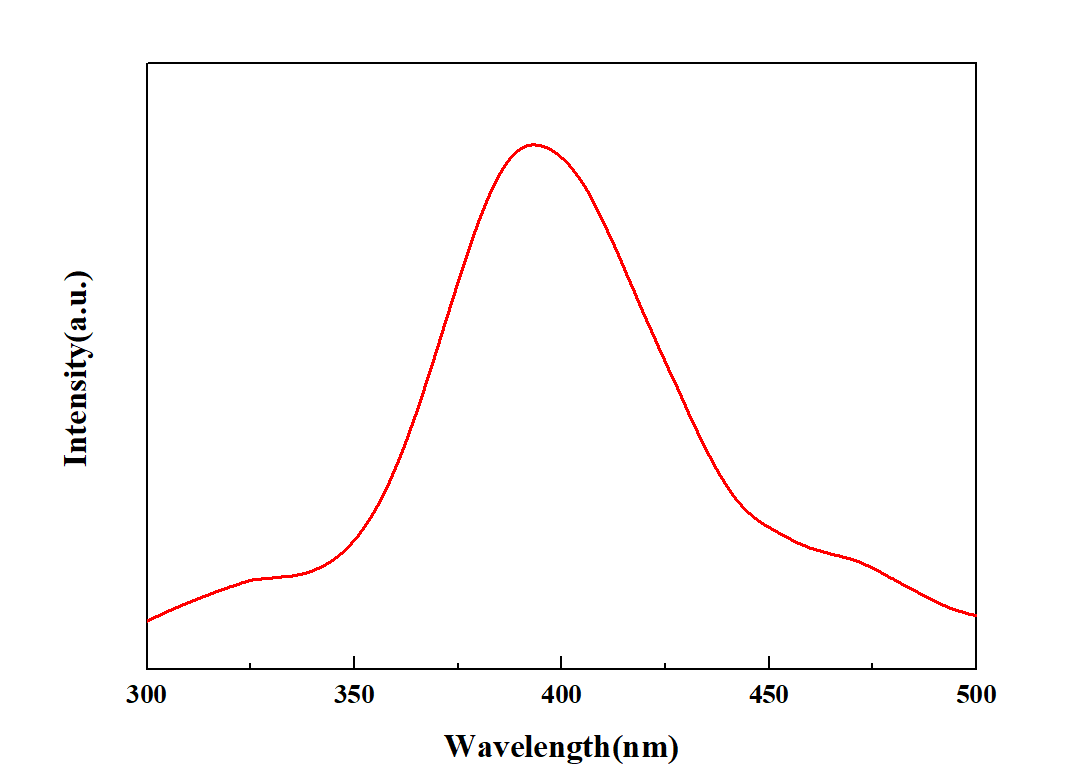
**

**Supplementary Figure 2. Photoluminescence spectrum for Au-Nb_3_O_7_F nanosheets upon excitation at λ = 246 nm at room temperature.**

**
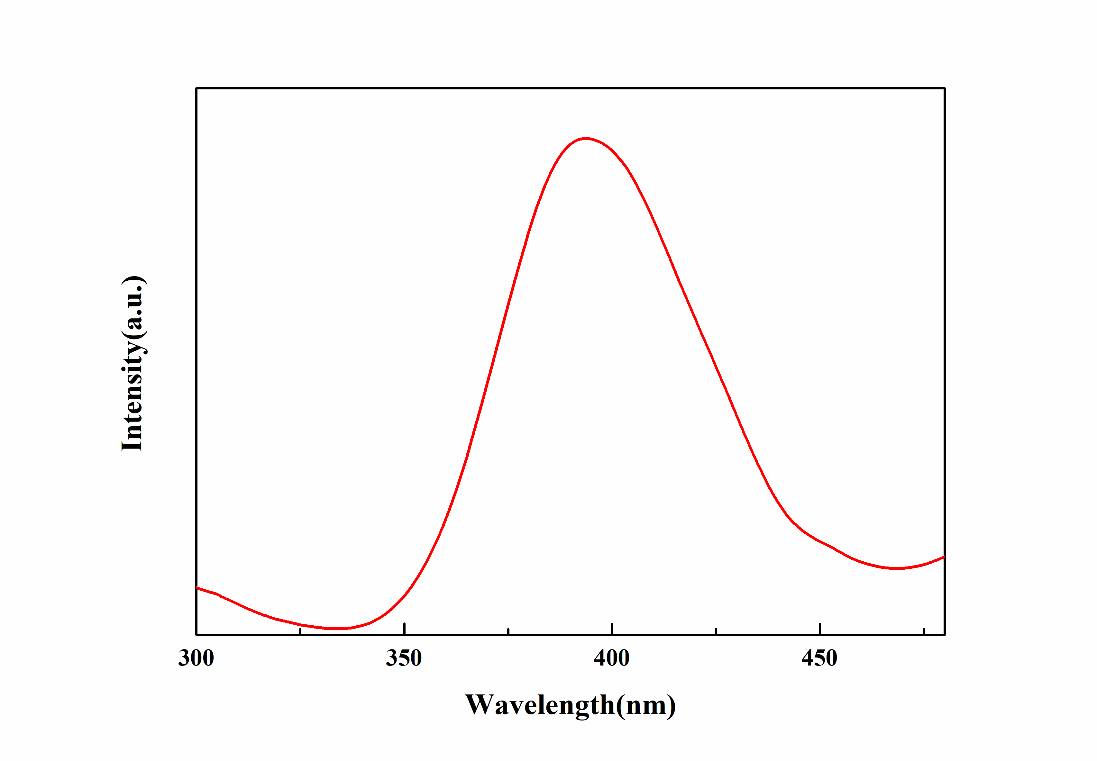
**

**Supplementary Figure 3. Photoluminescence spectrum of Nb_3_O_7_F nanosheets upon excitation at λ = 246 nm at room temperature.**

**III. HPLC of 0.4 Au-Nb_3_O_7_F degradation of TC-HCl**

HPLC analyses were carried out on a Waters E 2695 system. During the light irradiation, 3.5 mL of the solution was taken out every 10 min and centrifuged to separate the solid. The supernatants were then analysed by HPLC. Conditions for analytical HPLC resolution were optimized as follows: flow rate = 1.0 mL/min, λ = 356 nm, retention time: 6.5 min.

0 min:


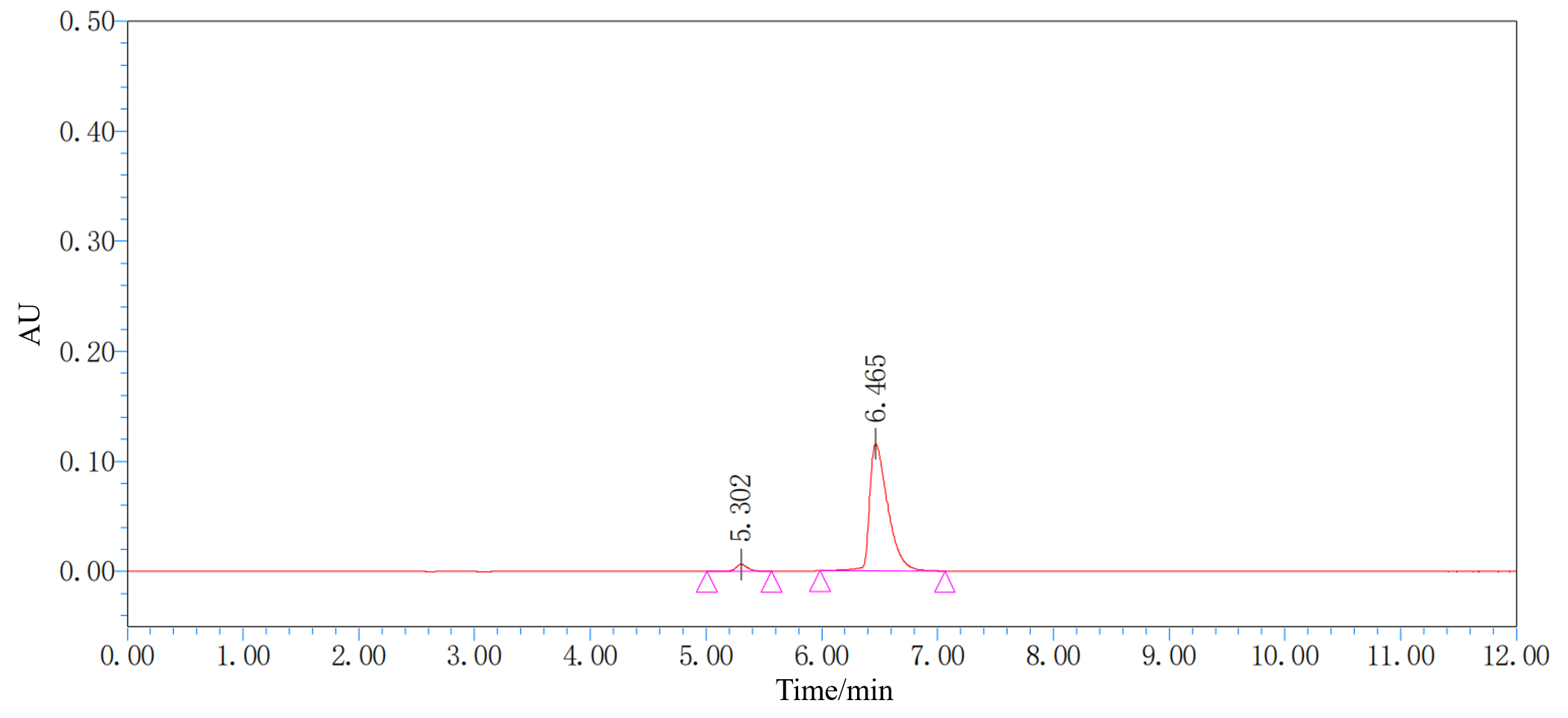


10 min:


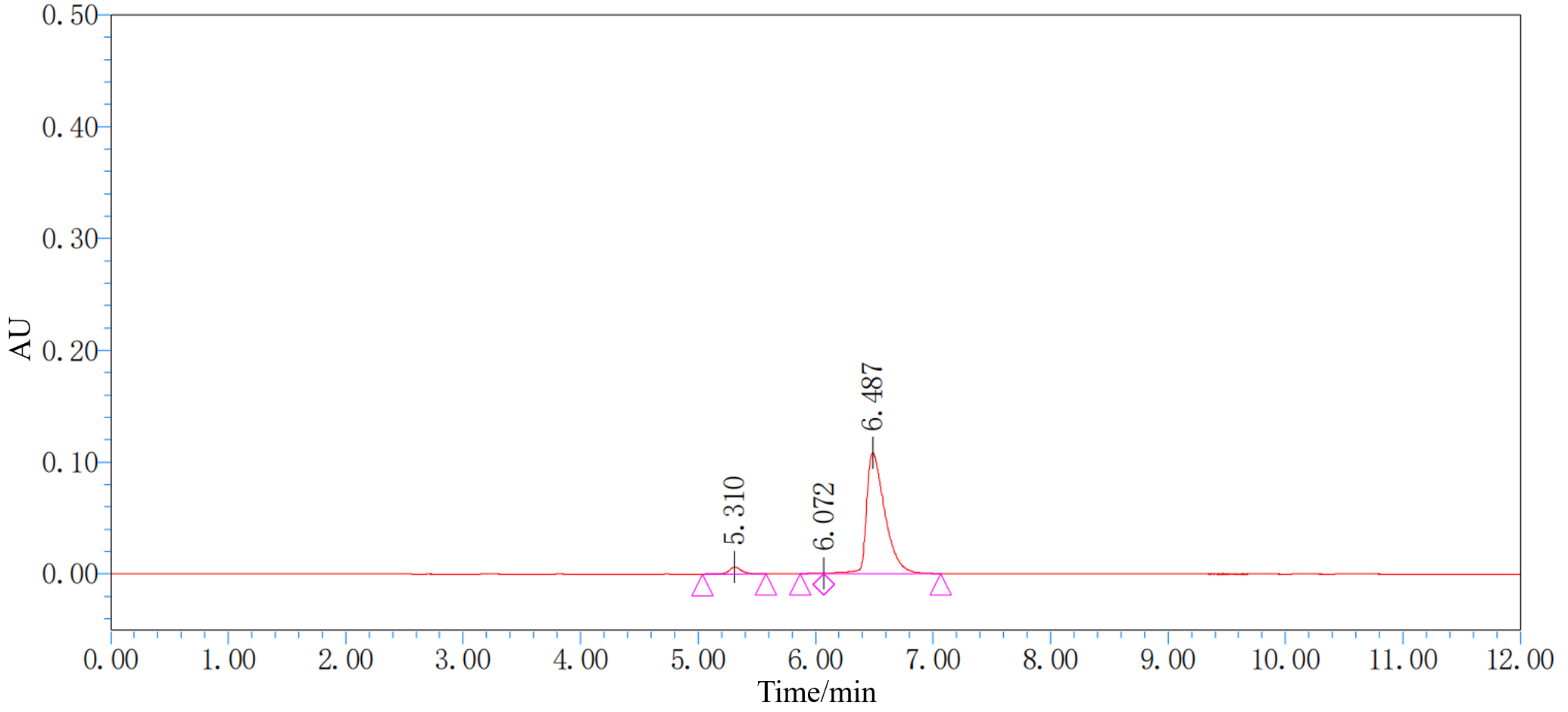


20 min:


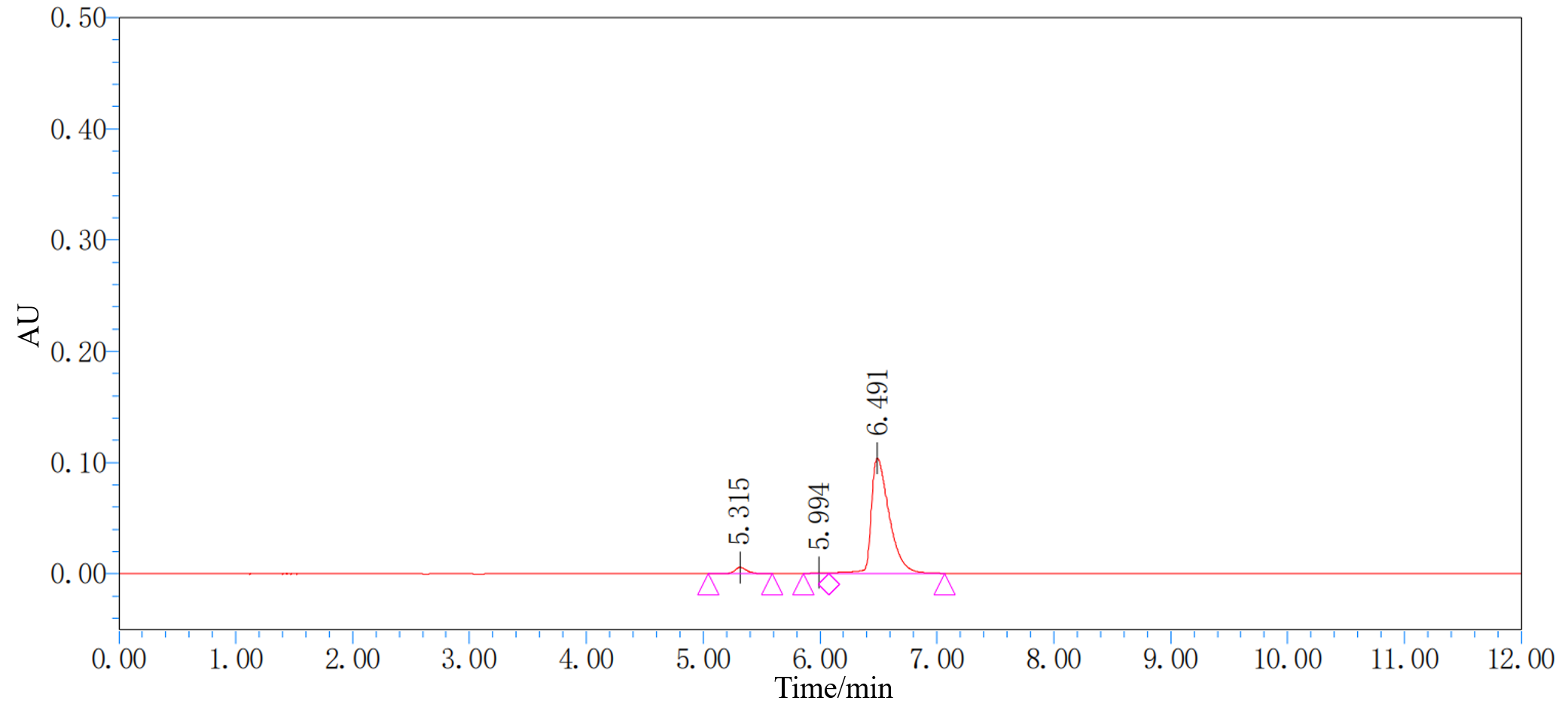


30 min:


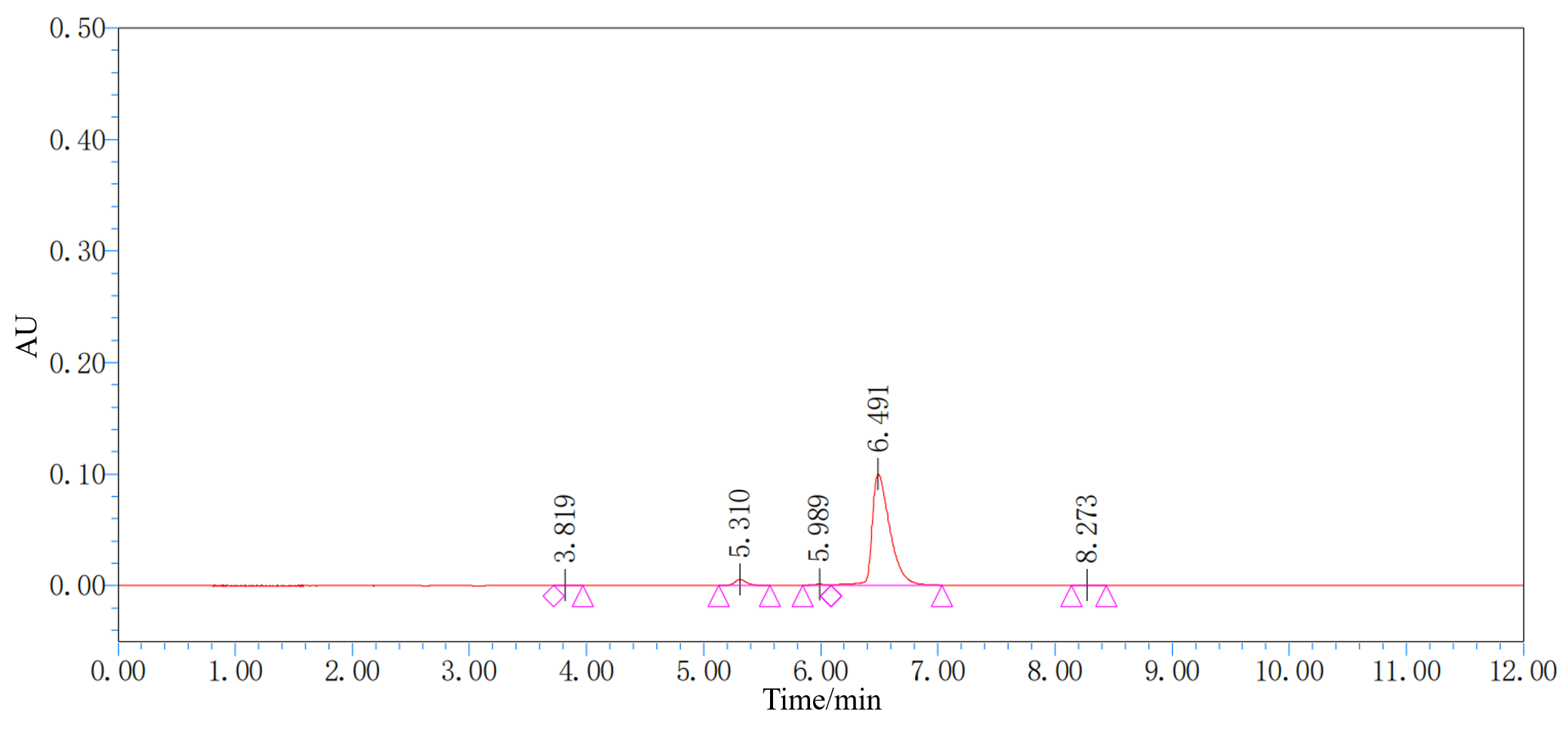


40 min:


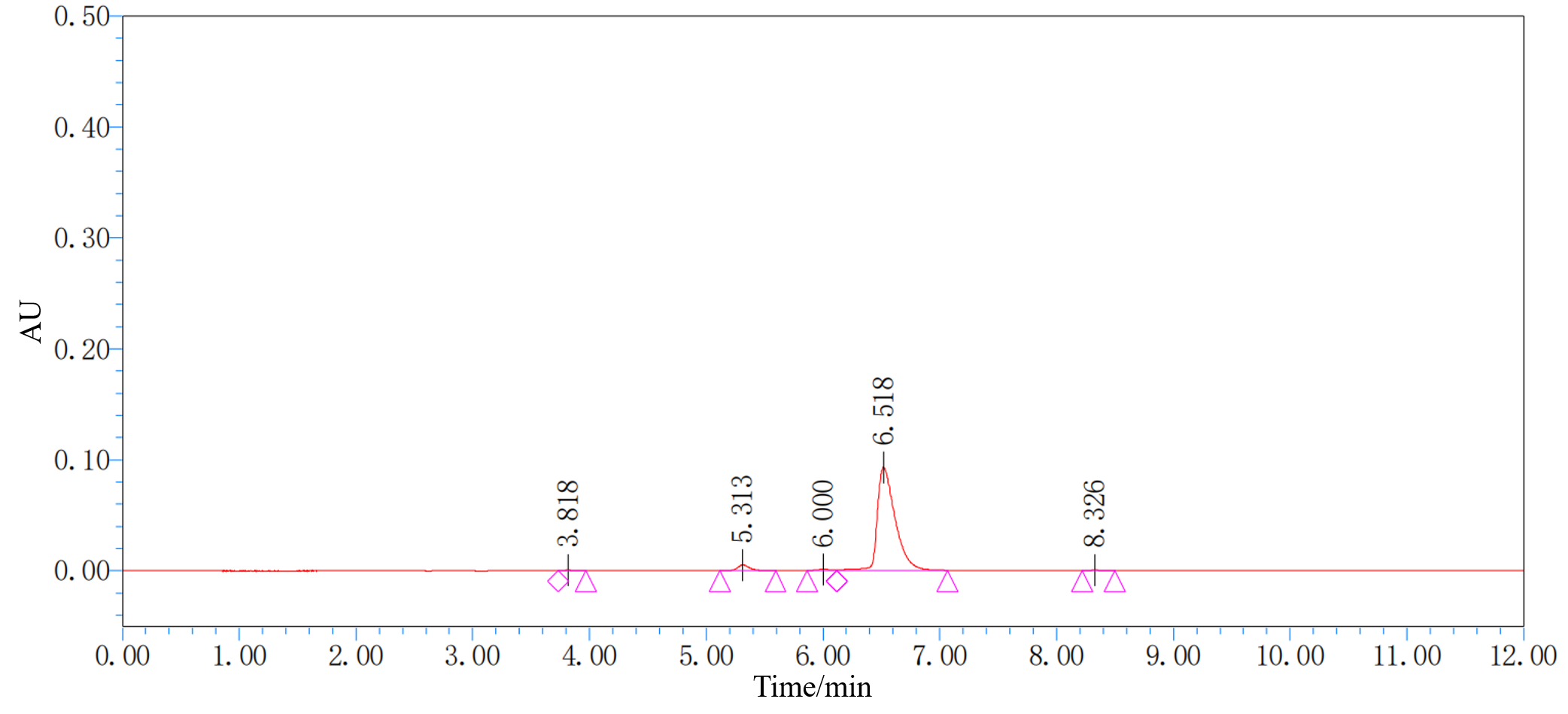


50 min:


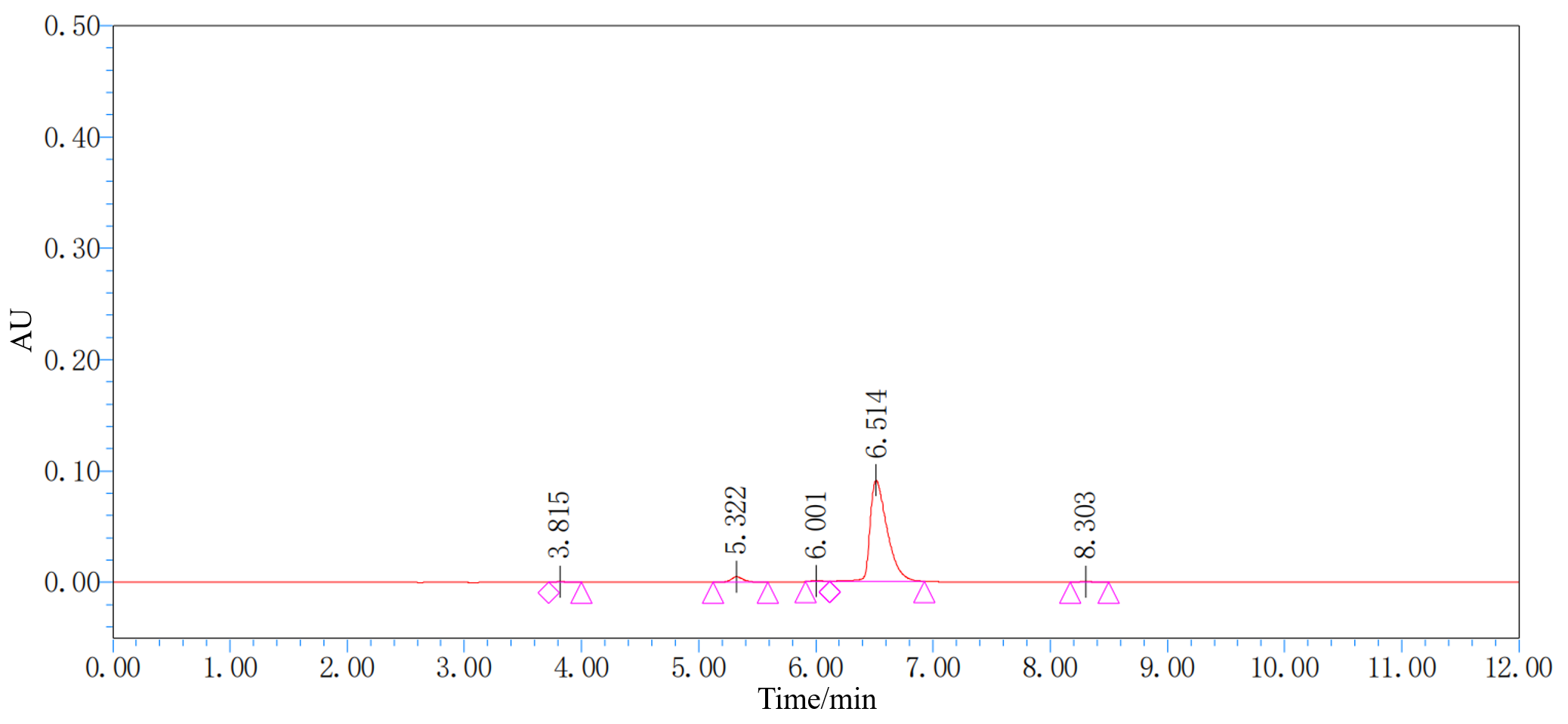


60 min:


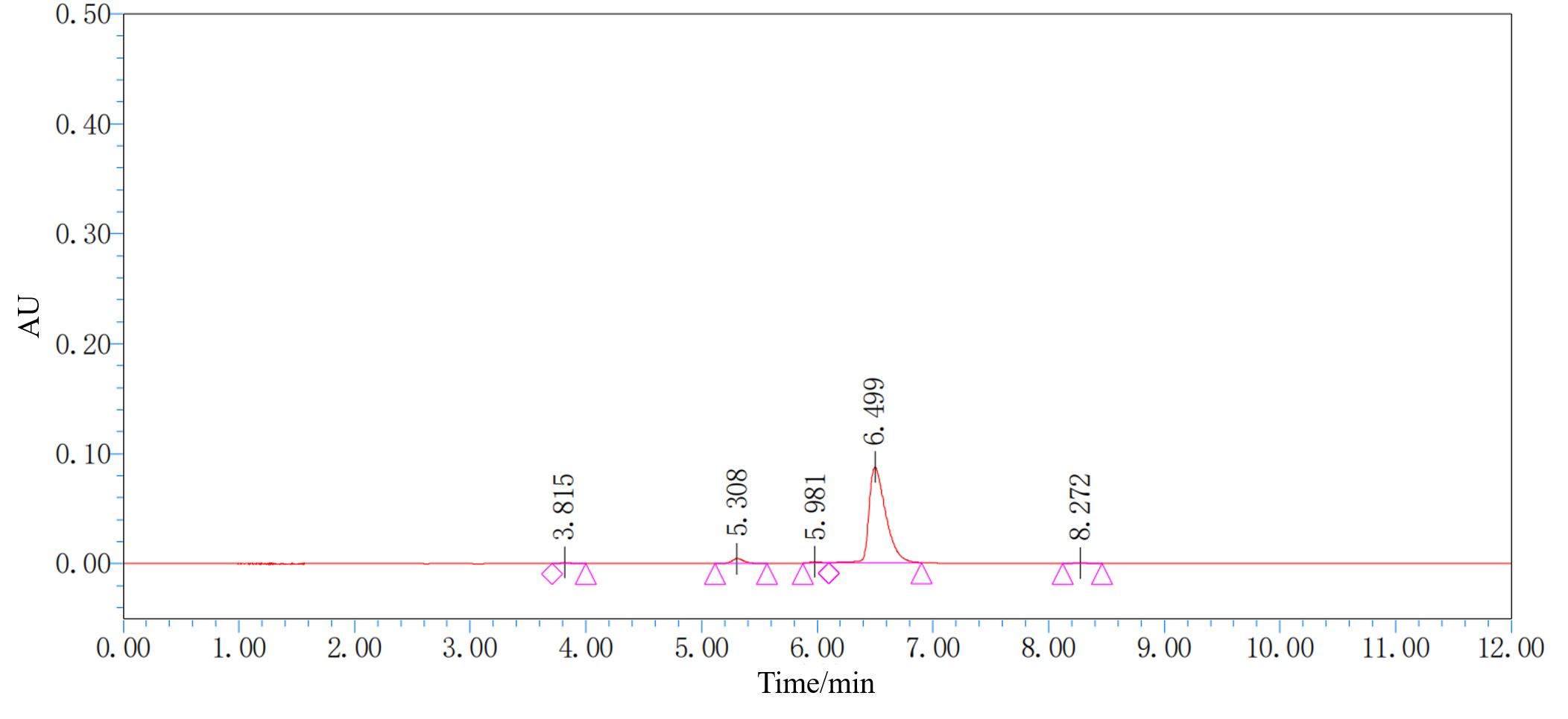


**IV. Recyclability of 0.4 Au-Nb_3_O_7_F nanosheets degradation of TC-HCl**


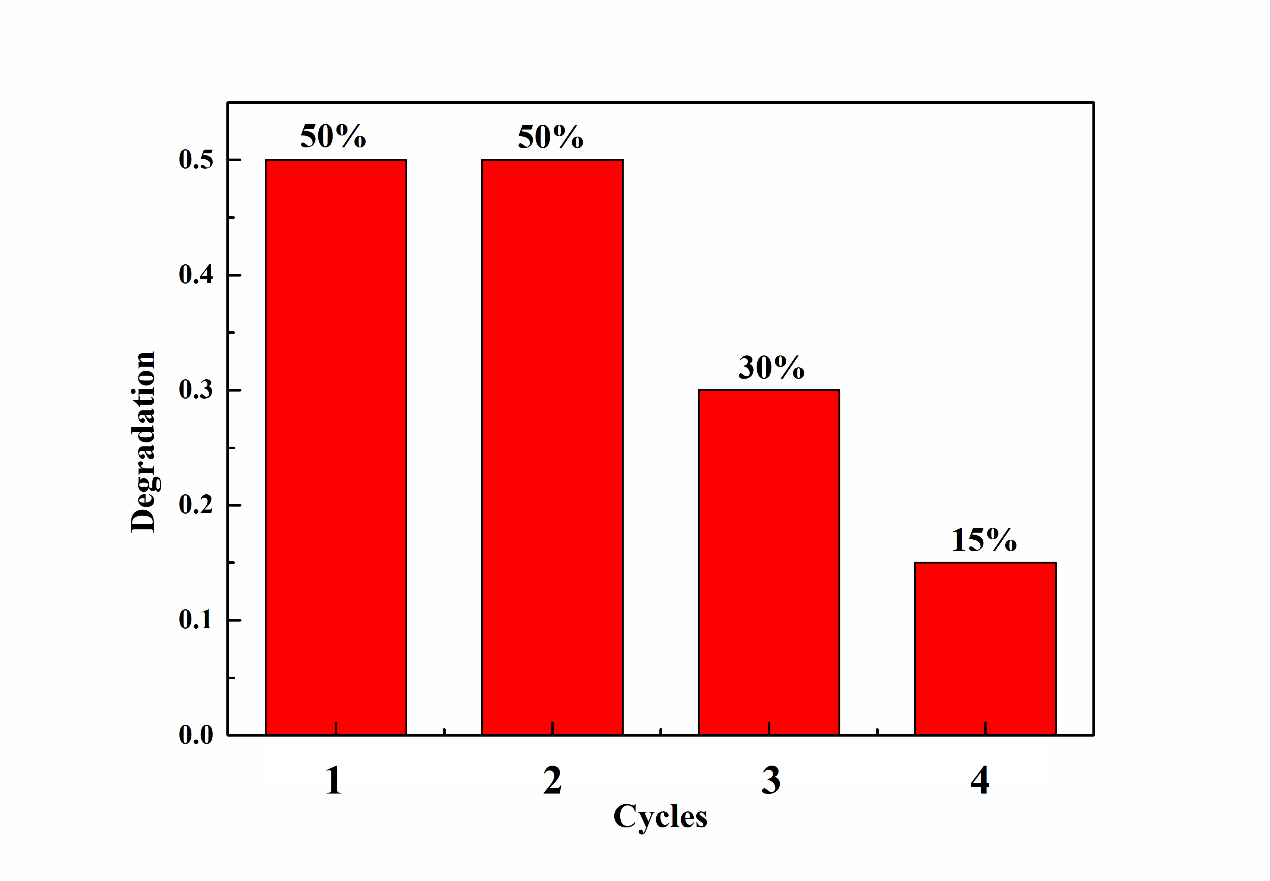


**Supplementary Figure 4.** Recyclability of 0.4 Au-Nb_3_O_7_F nanosheets degradation of TC-HCl


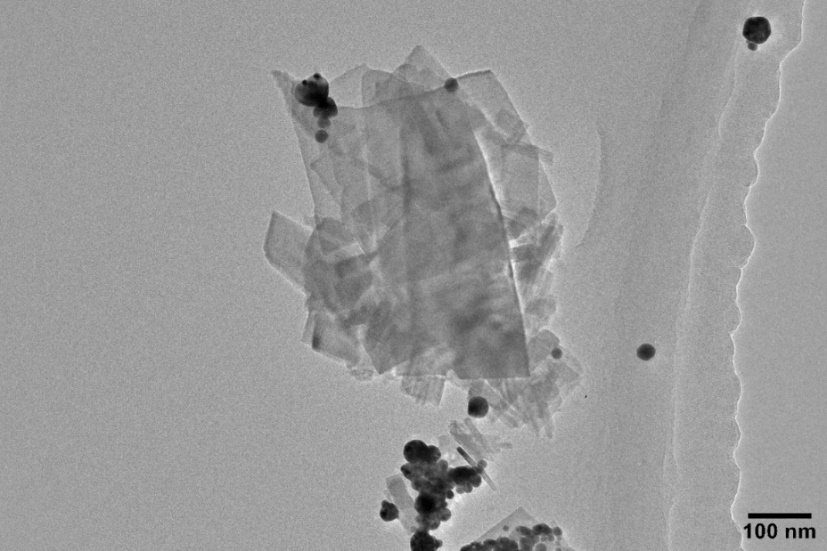


**Supplementary Figure 5.** TEM image of 0.4 Au-Nb_3_O_7_F nanosheets

after three cycles of TC-HCl degradation

**V. Photodegradation curves of tetracycline hydrochloride for 4 h**

**

**

**Supplementary Figure 6. Photodegradation versus time curves of tetracycline hydrochloride for 4 h**

**VI. Chemical composition of the Au/Nb_3_O_7_F composites**

**Supplementary Table 2. The chemical composition of the Au/Nb_3_O_7_F composites**

**determined from the XPS spectra**

| Name | Atomic % |
| --- | --- |
| Au | 1.44 |
| C | 35.04 |
| F | 6.13 |
| Nb | 14.32 |
| O | 43.07 |

**VII.** **Photodegradation curves of TC-HCl concerning the adsorption equilibrium**


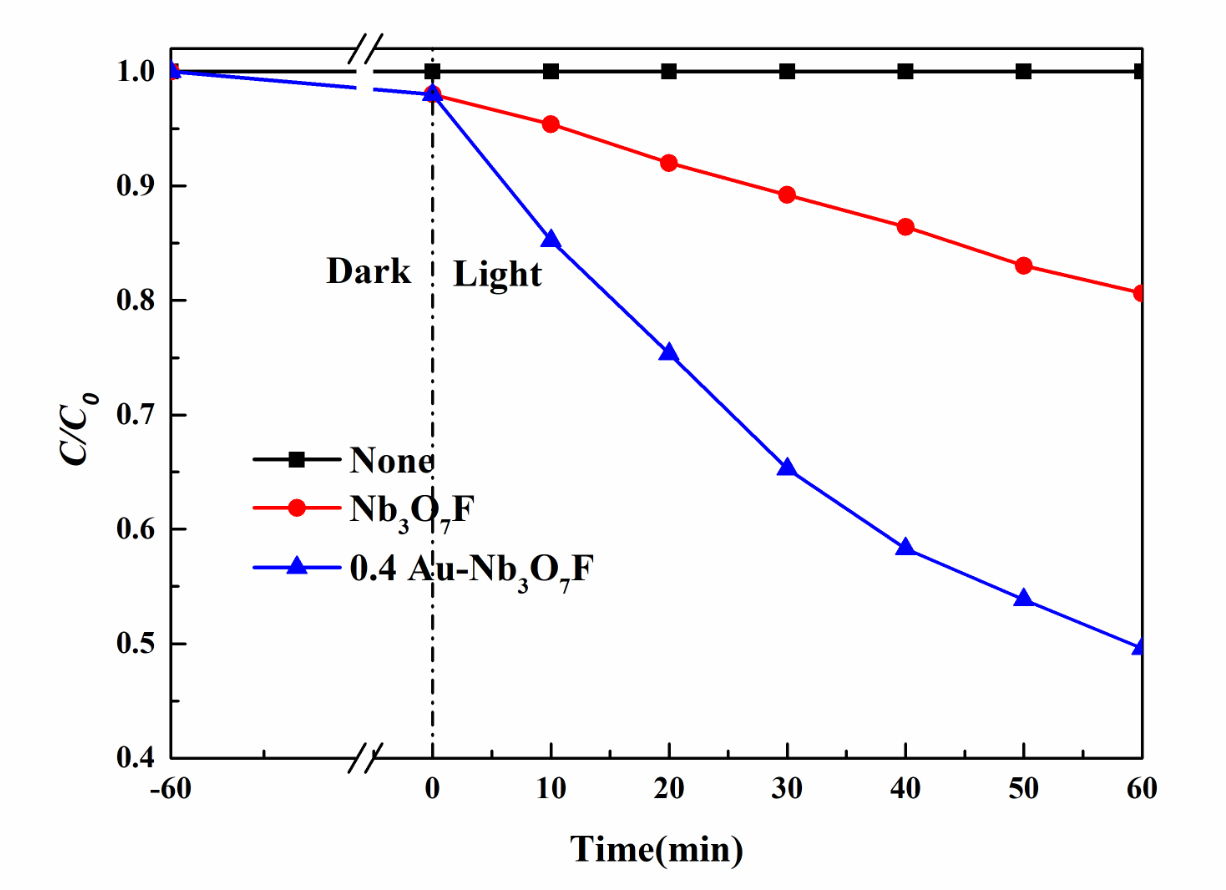


**Supplementary Figure 7. Photodegradation curves concerning the adsorption equilibrium**

**VIII. Tauc’s Plot**

**
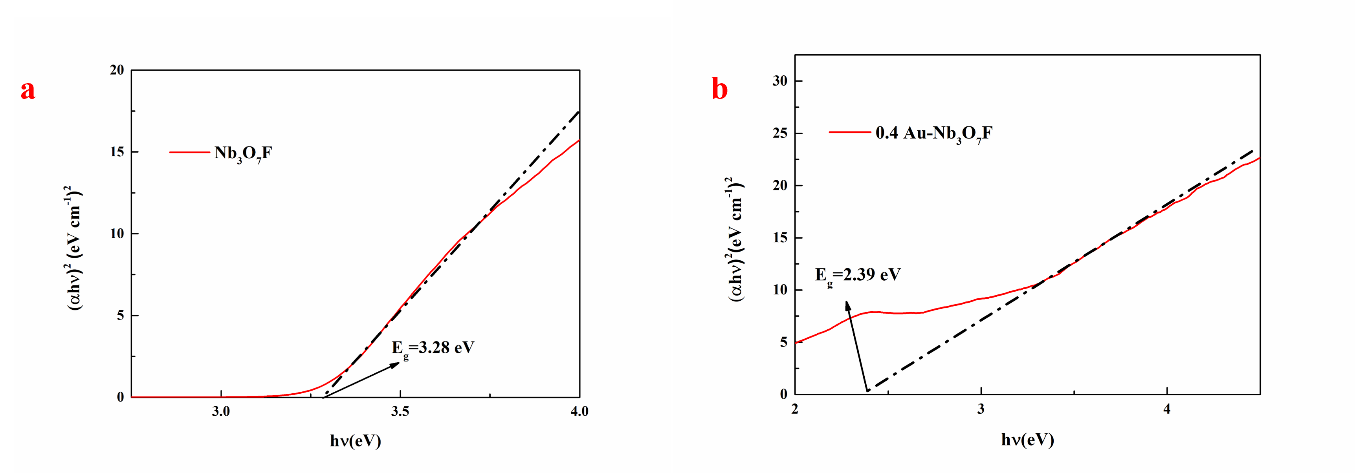
**

**Supplementary Figure 8**. **(a)** **bandgap of Nb_3_O_7_F; (b)** **bandgap of 0.4 Au-Nb_3_O_7_F.**
